# A real-time virtual liver simulator using position based dynamics with compliant constraints for medical applications

**DOI:** 10.1038/s41598-025-11519-4

**Published:** 2025-07-22

**Authors:** Yufan Guo, Dongliang Kou, Xukun Zhang, Lihua Zhang, Kai Cao

**Affiliations:** College of Intelligent Robotics and Advanced Manufacturing, Fudan University, Shanghai, China

**Keywords:** Computational science, Computer science, Information technology, Software, Medical research, Computational biology and bioinformatics, Computational platforms and environments

## Abstract

Liver simulation serves as a crucial foundation for precise preoperative planning and intraoperative navigation, with its accuracy and real-time performance directly impacting the safety and efficiency of surgical procedures. However, existing physical simulation techniques face a trade-off between high precision with slow computational speeds and rapid performance with diminished accuracy. This imbalance restricts their utility in dynamic surgical environments that demand both accuracy and real-time interaction. To overcome this limitation, we present a novel real-time liver simulation algorithm leveraging Extended Position-Based Dynamics (XPBD). Our approach integrates four well-formulated physical constraints–Distance, Volume, Shape Matching, and the Neo-Hookean model–within a proprietary, high-efficiency physics engine, achieving high-precision, real-time simulation of hepatic geometric deformations and mechanical properties. Experimental evaluations reveal that our method not only meets the stringent real-time requirements of surgical procedures but also significantly enhances the visual realism and stability of simulations. This advancement demonstrates substantial potential for extensive applications in preoperative planning, intraoperative navigation, and medical training.

## Introduction

The liver, the largest internal organ in the human body^1^, possesses a sophisticated geometric structure and unique biomechanical properties, making it a highly valuable subject for surgical clinical research. Its diseases, especially liver cancer, highlight the urgent need for early detection and effective intervention, as liver cancer is often diagnosed at an advanced stage due to its subtle early symptoms. Current diagnostic and therapeutic strategies are limited by the inability to model liver deformation in real time^2^. Advanced simulation techniques are crucial for improving preoperative planning, intraoperative navigation, and medical training, ultimately enhancing liver disease treatment.

Virtual Reality, Augmented Reality, and Physical Simulation technologies are increasingly pivotal in modern medicine, integrating fields such as Computer Graphics, Computer Vision, and Biomechanics. These technologies utilize advanced computational methods and sophisticated physical simulation algorithms to realistically mimic complex physical phenomena in the real world, providing a secure and controllable environment that enables practitioners to refine techniques and strategies without risk to patients, thereby improving surgical success rates and enhancing patient outcomes^3^. The application of physical simulation technology spans the entire spectrum from surgical procedures to the diagnosis, research, and treatment of liver diseases^4,5^. By integrating biomechanical models and imaging data, precise localization and qualitative or quantitative assessment of liver lesions can be achieved, providing scientific evidence to support clinical diagnosis and treatment decisions. In response to the challenges in liver disease treatment and surgical planning, such as the anatomical complexity and real-time requirements of traditional methods and the lack of adaptability to individualized patient data, we have developed a high-fidelity, real-time virtual liver simulator that holds significant clinical and practical value.

Our virtual liver simulation can rapidly and accurately replicate the geometric and mechanical properties of the liver. A real-time high-fidelity liver simulator not only improves surgical safety and efficacy but also finds extensive applications in various fields such as preoperative simulation, intraoperative navigation, ultrasound-guided biopsies, medical training, student education, liver registration, research of liver disease, training for robot-assisted surgery, evaluation and planning of liver transplants, and rehabilitation training and evaluation, all of which depend on real-time high-fidelity simulation technology^6–12^.

Physics-based soft body simulation plays a crucial role in enhancing realism in applications such as VR games, virtual surgery, and virtual cloth. Operations like deformation, cutting, and suturing are particularly challenging in surgical simulations, necessitating high-quality organ models. The essence of virtual physical simulation lies in creating mathematical models and employing physical simulation algorithms to accurately model and compute the physical behavior of objects, which typically involves solving complex partial differential equations to capture the dynamic response of objects under force and deformation. In computer graphics, these models are widely used in animations, special effects, and virtual reality systems to create highly realistic visual effects. However, the requirements are more stringent in biomedical simulations because biological tissues possess much more complex physical properties than inanimate objects. Biomechanical simulation focuses on simulating the physical behavior of biological tissues and organs, involving complex mechanical properties such as non-linearity, anisotropy, and viscoelasticity. This poses higher demands on the real-time performance and accuracy of modeling and computation. By combining physical simulation methods with computer graphics, these complex calculations are simplified while ensuring efficient and accurate simulation results. Integrating computer graphics with biomechanics, we have developed a new approach that incorporates robust constraints into XPBD to achieve visually realistic and real-time effects for virtual liver simulations, meeting medical application requirements.

- We are the first to perform an online, real-time, continuous simulation of the virtual liver using an XPBD physical simulation algorithm.
- We define four well-formulated physical constraints (Distance, Volume, Shape Matching, Neo-Hookean model) and Lagrange multipliers, adjusting parameters through Young’s modulus and Poisson’s ratio to closely approximate physical reality based on the biomechanical properties of the liver.
- We have self-developed a physical engine equipped with our liver simulator, enabling the complete pipeline from CT data to the final dynamic visualization of the liver, laying the groundwork for future operations and the realization of a universal, versatile physical engine for realistic liver simulation.

## Related work

With the continuous advancement of medical technology, the quality of soft tissue models for virtual surgery demands higher standards. Soft tissue models are now expected to reflect the real biomechanical properties of tissues and realistically simulate their mechanical behavior under force. This gave rise to physics-based models such as the Mass-Spring Model, Finite Element Method, and mesh-free models. Among these, the FEM treats soft tissue as a continuum based on solid mechanics, employing constitutive laws to describe complex mechanical behaviors. This method requires constructing spatial discretized meshes to approximate the governing laws of soft tissue mechanics. While it offers high simulation accuracy, its computational demands make it unsuitable for real-time surgical simulations involving large topological changes like cutting and suturing. FEM has been extensively studied for biomechanical analysis and simulation of biological soft tissues. Its applications range from head injuries^13^, orthognathic surgery^14^, and human hands with skin and muscle^15^, to the liver and blood vessels^16^. Implementations using the SOFA framework^17^ have been employed in procedures involving needle insertion into tissues^18^. Although FEM offers powerful modeling capabilities, achieving a balance between accuracy and computational speed remains challenging. However, FEM’s general strategy suffers from inefficiencies in explicitly applying positional constraints during simulations, leading to time-consuming precise results. This necessitates position-based methods for high-resolution real-time modeling.

Position-Based Dynamics (PBD) is a method that updates the positions of objects through iterative Gauss-Seidel processes to satisfy a series of constraints, such as collision detection and distance constraints, thereby achieving the simulation of object motion and deformation^19^. Due to its computational simplicity and ease of implementation, PBD has long been a research hotspot in the field of physics-based simulation in computer graphics, especially suitable for applications in game physics effects^20^. PBD models objects by manipulating positional displacements to resolve geometric constraints. Compared to force-based methods that achieve equilibrium configurations through acceleration integration, this geometric approach directly projects positions as solutions to quasi-static problems.

In PBD, an object is composed of multiple particles, and by manipulating the system’s constraint functions, various material properties and behaviors can be modeled. Shape Matching introduced a geometric constraint to simulate deformable objects suitable for game engines^21^. However, this method has a strong dependence on the iteration count’s stiffness and is limited to small deformations^22^. To accommodate larger motions, such as modeling soft tissues, supplementary cluster-based deformations can be integrated. The advantages of this type of implementation include robustness, simplicity, visual accuracy, real-time performance, efficiency, and controllability. Due to its real-time performance and visual capabilities, this geometry-driven, mesh-free concept is used in animation modeling in computer graphics, ensuring stable simulations while maintaining low computational time. A similar Gauss-Seidel constraint solver was proposed^23^. These methods operate under local constraints and are popular due to their efficiency and ease of implementation, though both depend on iteration counts for stiffness. PBD has been widely applied to simulate deformable objects. A continuum-based formulation was introduced that treats strain energy directly as a constraint function^24^. Additionally, terms for the Green-Saint Venant strain tensor were introduced to control strain independently of model discretization directions^25^. In contrast, our compatible constraint formulation directly corresponds to traditional constitutive models and converges to a well-defined solution.

PBD methods are also widely used in the medical field. Real-time surgical suture simulation has been developed for robust and interactive knot tying simulations^26^. The mass-spring model combined with shape matching techniques has been applied to achieve fast and stable simulations in virtual reality systems, focusing on heart model deformations^27^. Given the scope of this research in developing a robotic surgery simulation platform, all feedback is visual, prioritizing visual fidelity over precise deformation accuracy. However, accurate representations of complex physical phenomena can be achieved by modeling and optimizing simulation parameters or combining simulations with continuum-based formulations^24^.

The renowned open-source medical simulator iMSTK^28^ is also developed based on PBD. It has been used to simulate mitral and aortic valves as simple material models for fluids, facilitating interactive parameterization^29–31^. Furthermore, integrating mass-spring damping constraints into PBD has enabled the simulation of soft tissue deformations in laparoscopic cholecystectomy, addressing the dependency between deformation effects and iterative variables^32^. While PBD has advantages such as high computational efficiency, simplicity, and stability, it also has drawbacks in simulating soft tissue deformations. For instance, handling large-scale, high-resolution soft tissue models increases computational complexity and time consumption. Moreover, PBD has limitations in realism and fidelity, especially in complex deformation scenarios, where it may fail to capture fine details and behaviors of soft tissues. Therefore, we introduced XPBD^33^ to improve PBD constraints for better representation of physical phenomena. XPBD incorporates a multiplier to reduce the dependence on time steps and iteration counts for stiffness, while adding physical properties to the model. A physically meaningful model will make the results more consistent with the real world.

**Figure 1:**
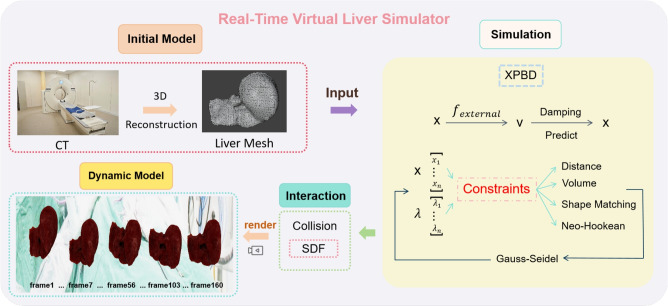
Pipeline of our simulator. The pipeline begins with clinical CT images reconstructed into initial liver meshes. Our advanced XPBD algorithm computes dynamic liver deformations, while a separate interaction module handles collisions. These processes are integrated to produce real-time animations of the liver model.

## Methods

### XPBD algorithm

We have integrated the XPBD algorithm into our self-developed physical simulation engine and the complete pipeline is shown in Fig. 1. For the virtual liver model, we define a set of *N* particles and *M* constraint functions. The subscript *i* denotes the iteration index, *j* is the constraint index. Specifically, the *i*-th $\in [1,\ldots , N]$ particle has a mass $m_i$, a position $\textbf{x}_i$ and velocity $\textbf{v}_i$. The constraints computed by $C_j$ ($j\in [1,\ldots , M]$) are applied to modify the position and velocity attributes of the *N* vertices at the next timestep $\Delta t$. The XPBD Simulation Process is described in Algorithm [1]. For simulation we start with a tetrahedral mesh as input and the force $f_{external}$ acts on each mesh. We create one particle for each vertex. Each tetrahedron adds one fourth of its mass to each adjacent particle.

**Algorithm 1:**
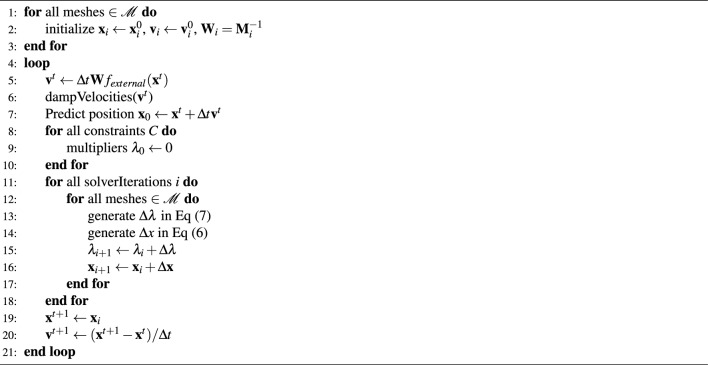
XPBD Simulation Process

The equations and inequalities representing the constraints can be unified into the form $C(\textbf{x}) \succ 0$, where the symbol $\succ 0$ denotes either $=$ or $\ge$. Consequently, the objective shifts to finding a correction $\Delta \textbf{x}$ that satisfies $C(\textbf{x}+\Delta \textbf{x}) \succ 0$. The nonlinear constraints can then be linearized as follows:

1 $$\begin{aligned} C(\textbf{x}+\Delta \textbf{x})=C(\textbf{x})+\nabla C(\textbf{x})\cdot \Delta \textbf{x}+O\left( |\Delta \textbf{x}|^2\right) \succ 0. \end{aligned}$$

The XPBD algorithm starts with Newton’s Second law of motion under forces derived from an energy potential $E_p(\textbf{x})$ in Eq. (2) and performs an implicit position-level time discretization of these equations, denoted by the time step index *t* in Eq. (3). Note that the gradient operator $\nabla$ is a row vector of partial derivatives:

2 $$\begin{aligned} & \textbf{f}_{elastic} = {\textbf {Ma}} = -\nabla _{\textbf{x}} E_p^T(\textbf{x}), \end{aligned}$$

3 $$\begin{aligned} & \textbf{M}\left( \frac{\textbf{x}^{t+1}-2\textbf{x}^t+\textbf{x}^{t-1}}{\Delta t^2}\right) =-\nabla _{\textbf{x}} E_p^T(\textbf{x}^{t+1}). \end{aligned}$$

The stiffness parameter $k_j$ defines the strength of the constraint in a range from zero to one. The energy potential, specified in terms of a vector of constraint functions, involves a block diagonal compliance matrix $\varvec{\alpha }$ corresponding to inverse stiffness ${\textbf {K}}$:

4 $$\begin{aligned} E_p(\textbf{x})=\frac{1}{2}\textbf{C}(\textbf{x})^T\varvec{\alpha }^{-1}\textbf{C}(\textbf{x}). \end{aligned}$$

we convert this to a compliant constraint formulation by decomposing the elastic force into its direction and scalar components using the Lagrange Multiplier^34^, where the vector of constraint multipliers $\varvec{\lambda }_{elastic}=[\lambda _1,\lambda _2,\cdots \lambda _m]^T$ is defined. Note that the time step from the Eq. (3) is incorporated into our compliance matrix by defining $\tilde{\varvec{\alpha }}=\frac{\alpha }{\Delta t^2}$ in Eq. (5):

5 $$\begin{aligned} \varvec{\lambda }_{elastic}=-\tilde{\varvec{\alpha }}^{-1}\textbf{C}(\textbf{x}). \end{aligned}$$

By substituting our expression for $\lambda$, we derive the discrete constrained equations of motion. To solve this non-linear system, we use a fixed-point iteration based on Newton’s method and simplify the computation by omitting geometric stiffness and constraint Hessian terms. These approximations connect our method with the XPBD, treating it as a quasi-Newton method. Ultimately, we derive an updated linear subproblem, streamlining the simulation process and enhancing efficiency, and the position update is subsequently determined by directly evaluating in Eq. (6):

6 $$\begin{aligned} \Delta \textbf{x}=\textbf{M}^{-1}\nabla \textbf{C}(\textbf{x}_{i})^{T}\Delta \varvec{\lambda }. \end{aligned}$$

We reconnect to XPBD by employing a Gauss-Seidel approach to solve our linear system. Equation (7) includes a reference to $\lambda _{ij}$, representing the total Lagrange multiplier for constraint *j* at the current iteration *i*, which must be stored and updated along with the system positions to ensure accurate constraint maintenance and dynamic response adjustments:

7 $$\begin{aligned} \Delta \lambda _j=\frac{-C_j(\textbf{x}_i)-\tilde{\alpha }_j\lambda _{ij}}{\nabla C_j\textbf{M}^{-1}\nabla C_j^T+\tilde{\alpha }_j}. \end{aligned}$$

### Constraints

(1) **Distance Constraint**: Firstly, let us consider the distance constraint function. The distance constraint between each set of connected particles ($\textbf{x}_1$ and $\textbf{x}_2$) can be satisfied by introducing:

8 $$\begin{aligned} C_\text {distance}{(\textbf{x}_1,\textbf{x}_2)}=|\textbf{x}_1-\textbf{x}_2|-d, \end{aligned}$$

where d is the initial distance indicated by rest spring length.

(2) **Volume Constraint**: The preservation of tetrahedron volume, represented by four corner particles $\textbf{x}_1,\textbf{x}_2,\textbf{x}_3,\textbf{x}_4$, can be kept constant by introducing:

9 $$\begin{aligned} C_\text {volume }(\mathbf {x_1},\mathbf {x_2},\mathbf {x_3},\mathbf {x_4})=\frac{1}{6}\left( \mathbf {x_{2,1}}\times \mathbf {x_{3,1}}\right) \cdot \mathbf {x_{4,1}}-V, \end{aligned}$$

where V is the initial volume of the tetrahedron.

(3) **Shape Matching**: Shape matching represents a geometrically driven technique for simulating deformable objects with an emphasis on rigidity preservation. The core concept entails dividing particles into various localized clusters. Subsequently, for each cluster, the objective is to identify the most suitable transformation that aligns the sets of particle positions before and after deformation, denoted as $\textbf{x}_i^0$ and $\textbf{x}_i$, respectively.

The goal function is to find the rotation matrix $\textbf{R}$ and the translation vectors $\textbf{T}$ and $\textbf{T}_0$ which minimize the error:

10 $$\begin{aligned} (\hat{\textbf{R}}, \hat{\textbf{T}}_0, \textbf{T}) = \operatorname {argmin}\left( \sum _i^n\Vert \textbf{R}\left( \textbf{x}_i^0-\textbf{T}_0\right) +\textbf{T}-\textbf{x}_i\Vert _2^2\right) . \end{aligned}$$

Here *n* denotes the total number of particles within the specified cluster. Detailed methodologies are elaborated in reference ^21^, which employs polar decomposition to analyze the transformation matrix. Consequently, the position adjustments for shape matching are calculated as:

11 $$\begin{aligned} \left[ \Delta \textbf{x}_i \right] _\text {shape matching} = \hat{\textbf{R}}(\textbf{x}_i^0-\hat{\textbf{T}}_0)+\hat{\textbf{T}}-\textbf{x}_i. \end{aligned}$$

(4) **Neo-Hookean model**:

There are many Neo-Hookean models. We use the simplest one with the elastic energy density in reference ^35^:

12 $$\begin{aligned} \Psi _{\textrm{Neo}}=\frac{\lambda }{2}\left( \det (\textbf{F})-1\right) ^2+\frac{\mu }{2}\left( \textrm{tr}(\textbf{F}^T\textbf{F})-3\right) =\Psi _\textrm{H}+\Psi _\textrm{D}, \end{aligned}$$

where the 3$\times$3 matrix *F* is the deformation gradient and $\lambda$ and $\mu$ the Lamé parameters. It has two separate energy terms, a hydrostatic energy $\Psi _\textrm{H}$ resisting compression and expansion and the deviatoric energy $\Psi _\textrm{D}$ resisting distortion. In accordance with the energy definition of the constraint function provided in Eq. (5), we present our hydrostatic energy $\Psi _\textrm{H}$ in terms of the following constraint function:

13 $$\begin{aligned} C_{\textrm{H}}(\textrm{F})=\det (\textrm{F})-1. \end{aligned}$$

To address the issue that compliant constraints can generally only represent non-negative energies, we vertically translate the Neo-Hookean energy into the positive half-space. Since forces are derived from the energy gradient, the forces resulting from this adjusted energy remain unaltered. The constraint function that generates this modified energy is:

14 $$\begin{aligned} C_{\textrm{D}}(\textbf{F})=\sqrt{\textrm{tr}\left( \textbf{F}^T\textbf{F}\right) }. \end{aligned}$$

We define four well-formulated physical constraints for simulation of the particle dynamics to generate the liver tissue deformation.

### Interactive dynamics engine

In our self-developed physics engine, we have developed and integrated a versatile module for collision detection and response applicable to triangular and tetrahedral meshes. The core functionality of this module utilizes the Signed Distance Function to calculate the minimum distance between objects, thereby determining the occurrence of collisions. Upon collision detection, this module employs a Continuous Collision Detection (CCD) algorithm to handle interactions among fast-moving objects, preventing penetration errors. Collision responses are precisely managed based on distance and normal information provided by the SDF, including calculating the direction of rebound, adjusting penetration depth, and appropriately modifying dynamic responses such as adjustments in velocity and angular momentum. Furthermore, we have integrated parameters such as damping, friction, and energy loss, and by finely tuning these parameters, we have further enhanced the realism and reliability of the simulations. To complement this, we have also developed and integrated an editable shader-based real-time rendering animation tool, enabling a seamless workflow from the initial model input to the final rendered output animation. It should be noted that all visual results and 3D visualizations presented in this study were generated using our self-developed physics engine.

## Results and discussion

### Dynamic response of liver to varying stiffness levels

Deformations are common in simulations of medical procedures, such as organ manipulation, surgical transection, and laparoscopic interventions. In this study, we used real-world patient CT scan data from the publicly available 3D-IRCADb-01. The liver data were three-dimensionally reconstructed using 3D Slicer software, and a volumetric tetrahedral mesh was generated using Delaunay triangulation in the CGAL library. All experiments were conducted using an NVIDIA GeForce RTX 4090 GPU. Our simulator dynamically adjusts the alpha of various constraints to model livers with different stiffness levels, accounting for variations due to age, population, and diseases like cirrhosis or fatty liver, which significantly alter liver stiffness. As liver stiffness is a critical biomechanical marker of disease progression, this visualization is invaluable for understanding and simulating disease impact.

However, conventional ultrasound presents several drawbacks in the preliminary diagnosis of liver fibrosis, including low sensitivity, subjectivity, and limitations in both qualitative and quantitative assessments. To address these issues, we used shear wave elastography (SWE), a non-invasive and effective method for quantitative data collection. SWE generates transverse shear waves via ultrasound excitation within the tissue, detected by ultra-fast imaging technology. The tissue’s elasticity is visualized in real time using color-coded maps, enabling the quantitative measurement of Young’s modulus and Poisson’s ratio. These parameters provide a direct, intuitive measure of tissue stiffness, facilitating the assessment of liver hardness and fibrosis extent. The formulas for Young’s modulus ***Ε*** and Poisson’s ratio ***ν*** are presented in Eqs. (15)–(16) by Lamé parameters, $\lambda$ and $\mu$:

15 $$\begin{aligned} & \lambda = \frac{E\nu }{(1 + \nu )(1 - 2\nu )}, \end{aligned}$$

16 $$\begin{aligned} & \mu = \frac{E}{2(1 + \nu )}. \end{aligned}$$

Young’s modulus quantifies the rigidity of a material, with higher values indicating greater resistance to deformation. An increase in Young’s modulus and decrease in Poisson’s ratio reflects the progressive stiffening of the liver, which correlates with the advancement of fibrosis. Quantitative measurements of liver stiffness across different individuals are typically expressed in kPa. Table 1 provides four data quantiles selected from publicly available detection results of diverse health populations. For clarity, the results are organized into four rows, each corresponding to a distinct stiffness configuration using Young’s modulus and Poisson’s ratio, we calculated compliance denoted as $\alpha$ to dynamically adjust four constraints in the physics engine.

**Table 1:** Experimental measurements of Young’s modulus and Poisson’s ratio from four representative participants with varying liver stiffness for real-time deformation simulations.

**Young’s modulus **(*E*) (kPa)	**Poisson’s ratio **($\nu$)
2.1972	0.4597
5.8597	0.4523
6.2781	0.4505
36.6175	0.4377

**Figure 2:**
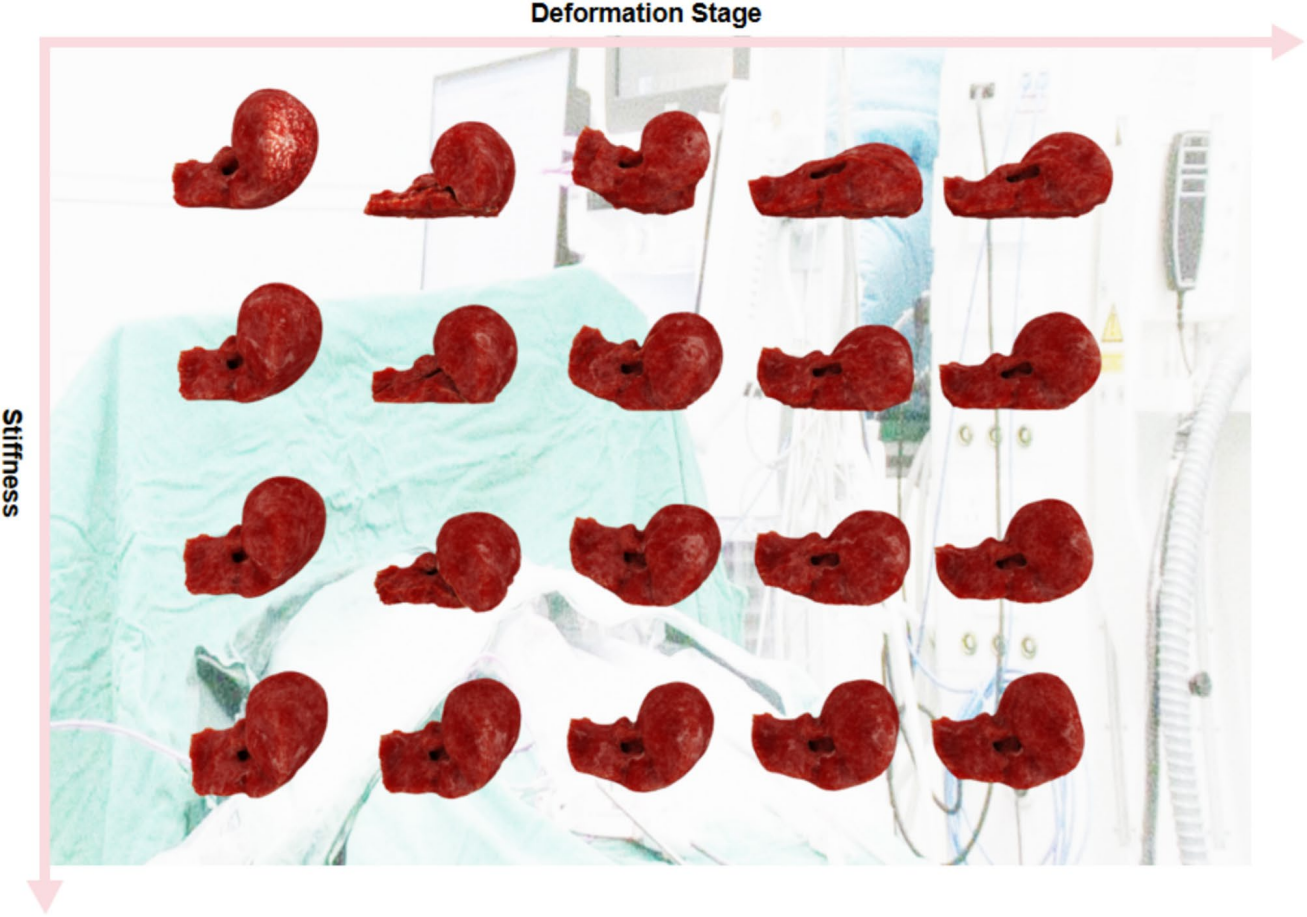
Deformation of the liver model under varying constraint conditions, as demonstrated in selected frames from our simulator. Each row systematically showcases a distinct stiffness configuration, ranging from more compliant (top row) to increasingly rigid (bottom row), highlighting the impact of these conditions on liver deformation.

Figure 2 illustrates the deformation process of the liver model under different constraint conditions. As part of our real-time virtual liver simulator, we captured frames during the experiment to display this process. Each column in the figure represents screenshots taken at different stages of the simulation. Each row in the figure represents a specific set of stiffness settings, ranging from softer (top row) to harder livers (bottom row). By gradually increasing the stiffness in the simulations, we observed variations in liver deformation under identical external forces. These external forces include gravity acting downward, friction with the ground, and elastic forces resulting from collisions. We defined the deformation task as the process in which the liver begins to deform from the moment it is subjected to external forces and collides with the ground, continuing until it returns to its original form without undergoing further significant deformation. Our experimental setup was designed to capture the dynamic changes in the liver when subjected to external forces, simulating physical interactions that could occur in real-life scenarios. Through detailed visual analysis, our study not only reveals the realism of the liver physical model but also provides valuable data supporting future biomechanically-based medical diagnosis and treatment planning.

Our engine not only integrates the physical constraints of the Neo-Hookean model but also allows for the substitution of other material models to customize the elasticity controls. Furthermore, the integration of Shape Matching constraints and the tunability of the Distance and Volume constraints enhance our model’s flexibility, extensibility, and fidelity, offering significant improvements over traditional PBD and FEM approaches. Our study also demonstrates the capability of constructing a liver simulator for medical visualization based on real patient data. The simulator achieves highly realistic physical and visual effects, aligning with the truth of computer graphics: if it looks right, it is right^36^.

### Comparative experiment

In our study, we specifically focused on comparing the real-time performance and visual realism of different simulation methods. We evaluated three distinct methods: traditional PBD, our liver simulator, and FEM on an identical liver mesh. The results shown in Table 2 record the average simulation times for handling the aforementioned defined deformation tasks and FPS during the runtime of the liver simulator. Notably, although our simulator recorded a slightly slower time than PBD, it significantly outperforms FEM in both runtime and FPS, clearly demonstrating its superior real-time performance capabilities. This marginal increase in processing time is justified by the significant enhancements in simulation complexity and physical realism offered by our simulator, establishing it as an effective solution for real-time medical simulation applications.

**Figure 3:**
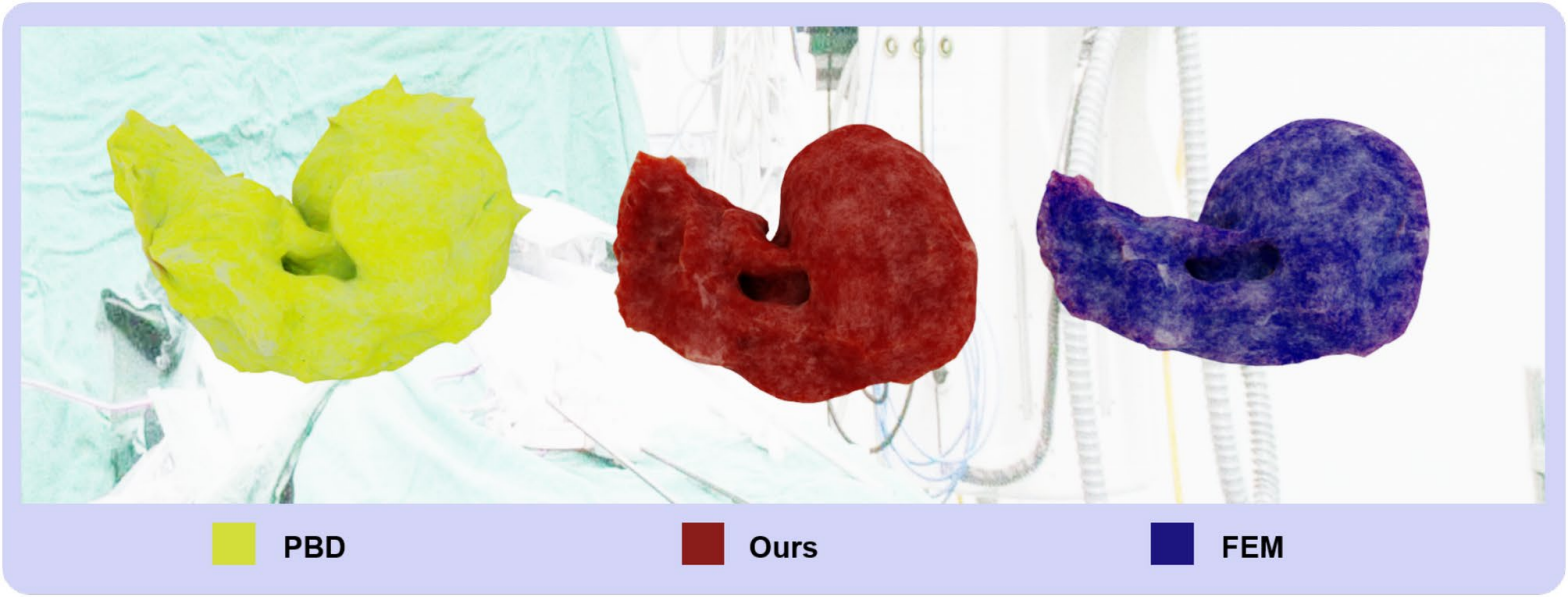
Visual Comparison of Liver Simulators: Sequential images from left to right show the simulation results generated by PBD (Yellow), Our Simulator (Red), and FEM (Blue).

**Figure 4:**
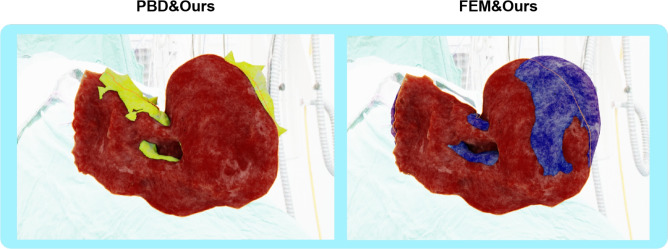
Striking Visual Comparison of Liver Simulators: Our simulator (outlined in orange) is overlaid with PBD (first image) and FEM (second image), clearly illustrating the differences in deformation characteristics at the point of maximum deformation on an identical liver mesh.

**Table 2:** Average simulation time (seconds) for handling aforementioned defined deformation tasks and comparative performance in average frames per second (FPS) of liver simulators with 64 substeps.

Method	Time	FPS
Position-based dynamics	1.03	194.8
**Our simulator**	**1.28**	**164.9**
Finite element method	7.49	26.1

XPBD incorporates advanced handling of physical properties, such as accurately simulating elasticity and dissipative energies. By introducing Lagrange multipliers and well-defined constraint functions, it ensures the fulfillment of positional constraints while precisely reflecting the physical responses of biological tissues like the liver, including elastic deformations and compressive characteristics. Figure 3 shows a visual comparison of liver simulators and in Fig. 4, our results, outlined in orange, are overlaid with PBD (first image) and FEM (second image) for clearer comparison. This overlay distinctly reveals that PBD produces unrealistic mesh protrusions and deformations caused by violent ground collisions, while FEM shows similar but slightly smaller deformations compared to our results, emphasizing the robustness and visual realism of our simulator. Unlike PBD, which lacks physical realism and does not incorporate physical properties, especially in handling complex biomechanical attributes such as the liver’s nonlinear elasticity and viscoelasticity, our simulator addresses these critical deficiencies. This added complexity is essential for achieving accurate results in medical simulations that demand high physical fidelity.

Severe mesh distortion in soft-body simulations frequently leads to non-convergent solutions, fundamentally revealing inherent limitations in numerical stability and precision when conventional simulation algorithms process large deformations. Although mitigation strategies such as Laplacian smoothing have been proposed, they incur critical trade-offs: iterative mesh smoothing inherently increases computational overhead and time consumption, with excessive iterations potentially introducing artificial model contraction. More importantly, such geometry-focused corrections neglect underlying physical motion characteristics, manifesting as numerical instabilities rooted in algorithmic precision limitations. To quantitatively evaluate mesh integrity, we define a smoothness metric *S* as:

17 $$\begin{aligned} S = \frac{1}{n} \sum _{i=1}^{n} \frac{V_{\min ,i}}{V_{\max ,i}} \end{aligned}$$

where *n* is the number of adjacent element pairs, and $V_{\min ,i}, V_{\max ,i}$ represent the smaller and larger volumes of adjacent tetrahedral elements, respectively. This dimensionless metric ranges from 0 to 1, with higher values indicating better volumetric consistency. Our simulator addresses these limitations through the integration of volumetric constraints and physical material model constraints. Conventional finite element methods often encounter similar instability challenges when they employ inappropriate constitutive models, particularly in complex biomechanical scenarios. Experimental validation confirms our methodology’s enhanced robustness over conventional approaches, establishing a reliable computational foundation for dynamic mesh models simulating anatomical deformations. Table 3 presents smoothness values across the three methods during maximum deformation. Our simulator demonstrates superior mesh integrity compared to both PBD and FEM approaches, with higher mean smoothness and lower standard deviation, indicating more consistent element volumes throughout the deformation process. This quantitative advantage directly correlates with the visual differences observed in Fig. 4, where PBD exhibits problematic mesh distortions during collision events.

**Table 3:** Comparative analysis of mesh smoothness at maximum deformation for different simulation methods using the same liver mesh.

Method	Average mesh smoothness	Standard deviation
Position-based dynamics	0.607	0.131
**Our simulator**	**0.786**	**0.067**
Finite element method	0.748	0.079

In contrast, while FEM offers slightly higher visual realism than PBD, its computational efficiency is lower, making it unsuitable for applications requiring real-time feedback. Our simulator achieves visual realism comparable to FEM while maintaining computational efficiency similar to PBD, effectively bridging the gap between these approaches. Therefore, our simulator is well-suited for use in virtual surgery and other scenarios that require both high-fidelity visualization and real-time feedback.Despite the advantages of our XPBD-based approach, it is important to acknowledge certain limitations specific to liver simulation applications. While XPBD effectively addresses the iteration count and time step dependency issues of traditional PBD^33^, our implementation still faces challenges in accurately modeling extreme anisotropic behavior that may be present in diseased liver tissue with significant fibrosis. Additionally, the accurate simulation of discontinuities that occur during surgical cutting procedures requires special handling beyond standard XPBD constraints, potentially necessitating adaptive remeshing techniques that could temporarily impact performance^37^. For scenarios involving complex fluid-structure interactions, such as blood flow within the liver’s vascular network, our current constraint system would require extension with specialized fluid handling capabilities. These limitations do not significantly impact the primary applications of preoperative planning and training, but represent important considerations for future development toward comprehensive surgical simulation environments. The extensible nature of our constraint-based framework provides a foundation for addressing these challenges while maintaining real-time performance.

### Applications and future prospects

The real-time virtual liver simulator significantly improves the accuracy of preoperative planning^38^ and intraoperative navigation^39^, especially in ultrasound-guided biopsies^40^, substantially improving surgeons’ ability to precisely locate complex liver structures, such as blood vessels and bile ducts. This optimization of surgical strategies reduces risks and increases success rates. Additionally, this technology provides a safe simulation environment in the field of medical education and professional training, allowing medical students and interns to improve their clinical operation skills through repeated practice. It also supports high-precision training for robot-assisted surgeries, helping surgeons master the skills needed to operate sophisticated robotic devices. Furthermore, liver simulation technology demonstrates its broad potential in applications such as liver transplant assessment and planning, as well as postoperative rehabilitation training and evaluation, all of which rely on accurate and real-time simulation capabilities. Particularly in telemedicine, this technology supports remote guidance and decision-making for complex surgeries by physicians in remote areas, enhancing surgical success rates and patient survival, especially valuable in resource-limited settings.

Building on this array of applications, our proposed methodology showcases substantial potential in liver simulations, highlighting the remarkable scalability and robustness of our virtual liver simulator. This scalability positions our work within the emerging paradigm of human body digital twins, which aims to create comprehensive digital representations of human physiology for personalized healthcare. A recent roadmap for developing human body digital twins describes a progression from isolated cross-sectional models to fully integrated physiological systems^41^. While pioneering efforts have focused on individual organs, such as GPU-accelerated digital twins of the human heart^42^ and advanced 3D reconstruction of tissues^43^, or specific physiological systems such as wearables-based prediction of motion intention through the nervous system^44^, the future lies in integrating these specialized components into comprehensive frameworks. Our liver simulator contributes a critical element to this larger vision, where multiple organ simulations could eventually be combined to model their interdependent relationships. As human body digital twin technology advances, our high-fidelity liver simulation methodology offers a valuable building block that could help bridge the gap between organ-specific models and fully integrated physiological systems. This approach is not only effective for liver simulations but is also adaptable for simulating other organs, such as the lungs^11^, heart^45^, and colon^46^. We are confident that this method is suitable for all organ simulations and broader medical scenarios, presenting a promising direction for future development. However, it also presents certain limitations; due to the unique biomechanical properties of different human organs, it necessitates the flexible selection of appropriate constraints to ensure that the simulations more accurately replicate the physical realities of the real world. In reality, there are even more complex scenarios. For instance, we have not yet considered situations where the liver may have tumors, causing significant changes in its properties and shape. This would complicate the boundary conditions, making them harder to manage. Extremely complex liver geometries and internal structures could also lead to potential inaccuracies in the simulator. The use of more specific and numerous constraints is among the areas where future improvements could be made.

Future research directions will further expand the application scope and enhance the precision and speed of liver simulation technologies. On one hand, surgical simulators integrated with AR have emerged as cutting-edge tools for surgical training and planning. This technology overlays virtual data onto actual operative environments, providing seamless visual and physical feedback to surgeons, thus enhancing surgical precision and efficiency, and allowing for the optimization of surgical strategies under conditions closely resembling actual scenarios. On the other hand, employing deep learning to optimize the solution processes of physical equations represents a critical direction for future development. By accelerating the computation of complex physical equations using deep learning algorithms, both simulation speed and accuracy are improved, reducing the reliance on high-performance hardware and facilitating the broader deployment of liver simulation technology across diverse medical environments. These research initiatives are anticipated to foster significant advancements in surgical planning, medical education, and telemedicine, thereby enhancing the quality and efficiency of global healthcare services.

## Conclusion

This study marks the first application of XPBD algorithm in liver model simulations, significantly advancing the field by enhancing the realism and immediacy of simulations. Our online, real-time, continuous simulator not only models biomechanical behaviors accurately but also adheres to the standards required for real-time processing, providing instantaneous feedback and adjustments for clinical applications across various liver textures. Additionally, within our proprietary physical simulation engine, we have implemented a range of functionalities and established four robust constraints: distance, volume, shape matching, and the Neo-Hookean model. These constraints endow the liver with realistic physical properties, setting new standards for physical simulations in medical practice.

The deployment of these technologies sets new benchmarks for the integration of physical simulations in medical practice, offering revolutionary tools for surgical planning and medical education. These tools enable highly precise real-time interactions for both students and professionals. Future research will focus on merging augmented reality technology and optimizing simulation processes with deep learning to further enhance the speed and accuracy of simulations. This progression is expected to extend its applications in remote healthcare and personalized medicine, promising to improve surgical precision, training efficiency, and patient outcomes globally.

## Data Availability

The data supporting the findings of this study are publicly available at https://www.ircad.fr/research/data-sets/liver-segmentation-3d-ircadb-01/.
